# Fluid Shear Stress Induces EMT of Circulating Tumor Cells via JNK Signaling in Favor of Their Survival during Hematogenous Dissemination

**DOI:** 10.3390/ijms21218115

**Published:** 2020-10-30

**Authors:** Ying Xin, Keming Li, Mo Yang, Youhua Tan

**Affiliations:** 1 The Hong Kong Polytechnic University Shenzhen Research Institute, Shenzhen 518000, China; ying-xy.xin@connect.polyu.hk (Y.X.); keminem20.li@connect.polyu.hk (K.L.); 2 Department of Biomedical Engineering, The Hong Kong Polytechnic University, Hong Kong 999077, China; mo.yang@polyu.edu.hk

**Keywords:** fluid shear stress, epithelial–mesenchymal transition, JNK, circulating tumor cells, metastasis

## Abstract

Tumor cells metastasize to distal organs mainly through hematogenous dissemination, where they experience considerable levels of fluid shear stress. Epithelial–mesenchymal transition (EMT) plays a critical role in tumor metastasis. However, how fluid shear stress influences the EMT phenotype of circulating tumor cells (CTCs) in suspension has not been fully understood. The role of shear-induced EMT in cell survival under blood shear flow remains unclear. This study shows that the majority of breast CTCs underwent apoptosis under shear flow and the surviving cells exhibited mesenchymal phenotype, suggesting that fluid shear stress induces EMT. Mechanistically, fluid shear stress-activated Jun N-terminal kinase (JNK) signaling, inhibition/activation of which suppressed/promoted the EMT phenotype. In particular, shear flow facilitated the JNK-dependent transition of epithelial CTCs into the mesenchymal status and maintained the pre-existing mesenchymal cells. Importantly, the induction of EMT suppressed the pro-apoptosis gene p53 upregulated modulator of apoptosis (PUMA) and enhanced the survival of suspended CTCs in fluid shear stress, which was rescued by overexpressing PUMA or silencing JNK signaling, suggesting that shear-induced EMT promotes CTC survival through PUMA downregulation and JNK activation. Further, the expressions of EMT markers and JUN were correlated with poor patient survival. In summary, our findings have demonstrated that fluid shear stress induces EMT in suspended CTCs via JNK signaling that promotes their survival in shear flow. This study thus unveils a new role of blood shear stress in CTC survival and facilitates the development of novel therapeutics against tumor metastasis.

## 1. Introduction

Cancer has become the second leading cause of human deaths globally, and over 90% of cancer-related deaths are attributed to metastatic dissemination. Metastasis is a complex and sequential process, which mainly includes cell detachment from the primary tumor, local invasion, intravasation into and survival in the vasculature, extravasation into distal organs, and formation of metastatic lesions [1]. Tumor cells metastasize to distal organs mainly through hematogenous dissemination. Therefore, the survival of circulating tumor cells (CTCs) in the vasculature is critical for the efficiency of the entire metastasis process. Many pre-clinical and clinical findings have demonstrated that the presence and frequency of CTCs in peripheral blood are correlated with the occurrence rate of tumor metastasis and patient survival [2,3]. After exiting the protective primary tumor microenvironment and entering the vasculature, CTCs become suspended and vulnerable to various factors in blood circulation. However, there still exists a small subpopulation of CTCs that can survive hematogenous dissemination and eventually generate metastases in distal organs [1]. It is thus essential to unveil the survival mechanisms of tumor cells during blood circulation for the effective prevention of metastasis.

Despite the importance of various biochemical factors in CTC survival and metastasis [4], cells have the ability to perceive mechanical stimulations, which can be converted into biochemical signals via mechanotransduction [5]. Accumulating evidence has demonstrated the important roles of mechanical factors in tumor metastasis [6], including the significance of blood shear stress in the viability and functions of tumor cells that are anchored to solid substrates. High levels of shear flow induce the production of reactive oxygen species and mediate cell damage [7]. Shear flow promotes cancer cell invasion by increasing the secretion of matrix metalloproteinases [8]. Fluid shear stress facilitates epithelial–mesenchymal transition (EMT) of adherent tumor cells, a process by which epithelial cells transit towards the state of mesenchymal cells, and enhances cell motility and cancer stem cell (CSC) properties [9]. In contrast, the effects of fluid shear stress on tumor cells in suspension remain less understood. Shear stress sensitizes suspended colon and prostate tumor cells to apoptosis [10]. High fluid shear stress induces substantial tumor cell apoptosis [11]. Therefore, fluid shear stress significantly affects tumor cell functions, including CTC apoptosis. Limited findings report the possible mechanisms of CTCs surviving hemodynamic shear stress, including Lamin A/C [12], actomyosin [13,14], and Atonal BHLH Transcription Factor 8 (ATOH8) [15].

EMT has been believed to influence tumor progression critically [1,16,17]. Clinical findings show that EMT is associated with tumor metastasis and poor patient survival [18,19,20]. EMT has been demonstrated to facilitate tumor cell survival [21,22] and confer CSC properties to conventional tumor cells [23]. Apart from many biochemical signalings, mechanical stimuli could also affect the EMT process. Elevated matrix rigidity facilitates EMT and promotes tumor metastasis [24,25,26]. Tumor growth-induced solid stress enhances EMT of renal cell carcinoma [27]. Fluid shear stress induces EMT in tumor cells, and tumor spheres adhered to substrates [9,28] and tumor cells in suspension [29,30]. However, how fluid shear stress influences the EMT phenotype of suspended CTCs has not been fully understood. In particular, how the shear-induced EMT changes affect CTC survival in blood shear flow remains unclear.

In this study, an in vitro circulatory system was adopted to generate physiologic levels of fluid shear stress, which mimicked blood shear flow in the vasculature. Tumor cells were exposed to various fluid shear stresses in suspension when cell viability and EMT phenotype were examined. A mechanistic study was conducted to explore the mechanism underlying shear-mediated EMT, especially c-Jun N-terminal kinase (JNK) signaling. Further, the roles of shear-induced EMT and JNK signaling in the survival of CTCs in fluid shear flow were investigated. In addition, the correlation between EMT and JNK signaling with patient survival was analyzed.

## 2. Results

### 2.1. Fluid Shear Stress Eradicates the Majority of Suspended Circulating Tumor Cells (CTCs)

**Figure 1:**
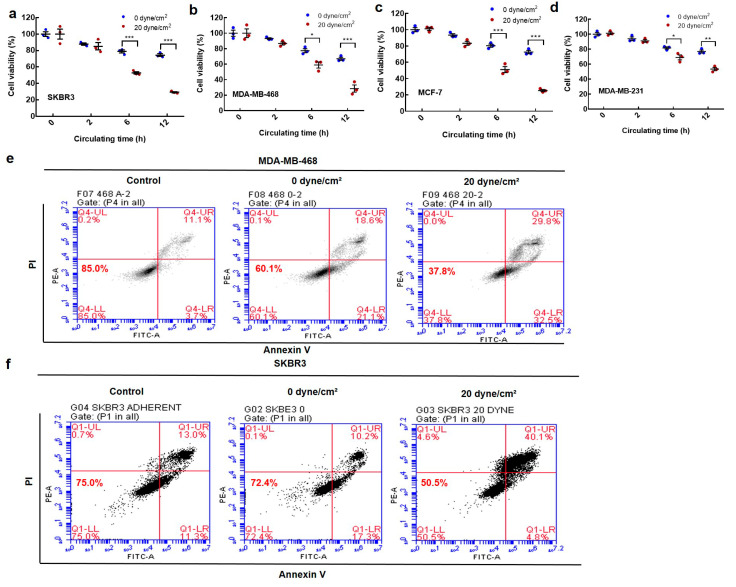
The influence of fluid shear stress on the viability of suspended tumor cells. (**a**–**d**) The viability of breast cancer cells in suspension. Single-cell suspensions of SKBR3 (**a**), MDA-MB-468 (**b**), MCF-7 (**c**), and MDA-MB-231 (**d**) were circulated under 0 and 20 dyne/cm^2^ shear stress for various circulation durations. Cell viability was measured by (3-(4,5-dimethylthiazol-2-yl)-5-(3-carboxymethoxyphenyl)-2-(4-sulfophenyl)-2H-tetrazolium) (MTS) assay and normalized by the data at 0 h under the same treatment. *n* = 3 independent experiments; (**e**,**f**) The influence of fluid shear stress on tumor cell survival. Cell apoptosis of MDA-MB-468 (**e**) and SKBR3 (**f**) cells was examined by the Annexin V-Fluorescein isothiocyanate (FITC)/Propidium iodide (PI) assay after circulation under 0 and 20 dynes/cm^2^ shear stress for 12 h. *n* = 2 independent experiments. * *p* < 0.05; ** *p* < 0.01; *** *p* < 0.001.

Tumor cells experience considerable levels of fluid shear stress during hematogenous dissemination. We have developed an in vitro system previously to simulate blood shear flow (Figure S1) [13]. The average shear stress within venous and arterial circulation is 0.5–4 dynes/cm^2^ and 4–30 dynes/cm^2^, respectively [31]. The duration of CTCs in the vascular system is within 12 h [32,33]. Therefore, fluid shear stress within 20 dyne/cm^2^ and the circulation time within 12 h were adopted in this study. To explore the influence of fluid shear stress on the viability of suspended tumor cells, breast cancer cells with different malignancy were circulated under various shear stresses for different durations. The results show that the viability of suspended CTCs gradually decreased under a designated shear stress along with the circulating time (Figure 1a–d). Cell survival was much lower under 20 dyne/cm^2^ than 0 dyne/cm^2^ shear stress at various time points. These findings were further confirmed using the Annexin V assay (Figure 1e,f). Similar phenomena were observed in four types of breast cancer cell lines (SKBR3, MCF-7, MDA-MB-468, and MDA-MB-231; Figure 1). These results suggest that fluid shear stress eliminates the majority of suspended CTCs.

### 2.2. Fluid Shear Stress Facilitates Epithelial–Mesenchymal Transition (EMT) in Suspended CTCs

**Figure 2:**
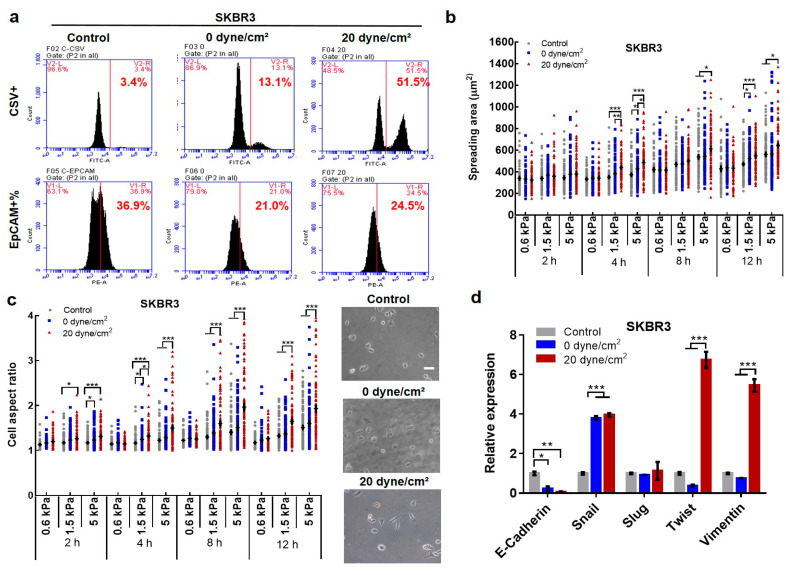

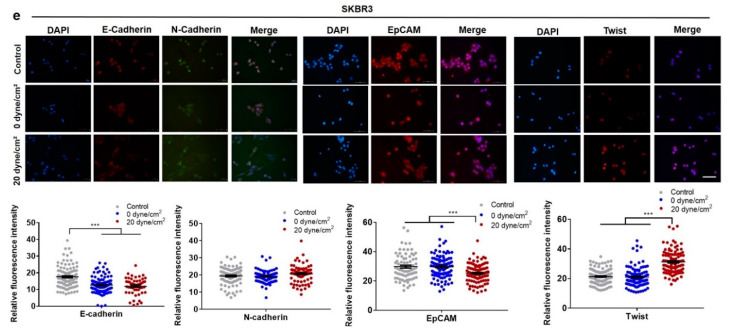
Fluid shear stress promotes the epithelial–mesenchymal transition (EMT) phenotype of suspended tumor cells. (**a**) Fluid shear stress enhances the cell-surface vimentin + (CSV+) fraction while reduces the epithelial cell adhesion molecule + (EpCAM+) fraction. Suspended tumor cells SKBR3 were treated under 0 and 20 dyne/cm^2^ shear stress for 12 h. The percentages of CSV+ and EpCAM+ cells were analyzed by flow cytometry. *n* = 2 independent experiments; (**b**,**c**) Fluid shear stress induces cell spreading and elongated morphology. The treated SKBR3 cells were cultured on 0.6, 1.5, and 5 kPa polyacrylamide gels. Cell images were taken at 2, 4, 8, and 12 h, respectively. The spreading area and cell aspect ratio were calculated; (**d**) Fluid shear flow upregulated/downregulated the expressions of mesenchymal/epithelial genes. *n* = 3; (**e**) Fluid shear stress enhanced/decreased the expressions of mesenchymal/epithelial genes at the protein level. Breast cancer cells were treated similarly as in (**a**,**d**). The expressions of E-cadherin, EpCAM, N-cadherin, and Twist were examined through immunofluorescence. The nucleus was counterstained with 4′6-diamidino-2-phenylindole (DAPI). Scale bar in (**c**,**e**): 50 µm. At least 100 cells were measured for each condition. The statistics were conducted using Analysis of variance (ANOVA) with the post hoc Bonferroni test in (**b**–**e**). * *p* < 0.05; ** *p* < 0.01; *** *p* < 0.001.

We have demonstrated that the majority of suspended tumor cells can be eliminated by fluid shear stress in blood circulation. However, a subpopulation of CTCs persists and exhibits resistance to shear flow, which may harbor the cells with the ability to eventually generate metastatic tumors. Since EMT is an evolutionarily conservative developmental program and highly involved in tumor metastasis [17], we thus examined the phenotype of the surviving CTCs after shear stress treatment. The cell-surface vimentin (CSV) was adopted to mark tumor cells with a mesenchymal phenotype [34], while epithelial cell adhesion molecule (EpCAM) was used to label tumor cells with an epithelial phenotype [35,36]. The results show that the percentage of CSV+ subpopulation was increased from 3.4% in untreated SKBR3 cells to ~13% after the treatment of 0 dyne/cm^2^ shear stress (Figure 2a). Remarkably, this fraction was elevated to ~51% in suspended CTCs after the treatment of 20 dyne/cm^2^ shear stress. Accordingly, the percentage of EpCAM+ subpopulation was reduced from ~37% in control cells to ~21% and ~24% after the treatment of 0 and 20 dyne/cm^2^ shear stress, respectively (Figure 2a). Note that the EpCAM+ fraction was slightly increased under higher shear stress. Similar findings were observed in another two breast cancer cell lines MDA-MB-468 and MCF-7 (Figure S2a,b), while the percentage of EpCAM+ fraction was reduced mildly in MDA-MB-468 cells and not even decreased in MCF-7 cells in response to shear stress (Figure S2b). The analysis of cell morphology showed that CTCs after shear treatment exhibited higher levels of spreading and more elongated cell shape than control cells (Figure 2b,c), reminiscent of the EMT phenotype. Further, the epithelial gene E-cadherin was markedly downregulated, while the mesenchymal genes were significantly upregulated (Figure 2d and Figure S2c,d). Note that there was no significant difference in Slug after the shear treatment and in Snail expression between 0 and 20 dyne/cm^2^ shear stress. The expression of Twist was decreased under 0 dyne/cm^2^ shear stress compared to control cells. The analysis at the protein level showed that the expressions of epithelial markers E-cadherin and EpCAM were decreased, while the expression of Twist was significantly increased after shear stress treatment (Figure 2e and Figure S2e,f). These data suggest that fluid shear stress facilitates the EMT process in suspended CTCs.

### 2.3. Shear-Induced EMT Depends on the Activation of Jun N-Terminal Kinase (JNK) Signaling

**Figure 3:**
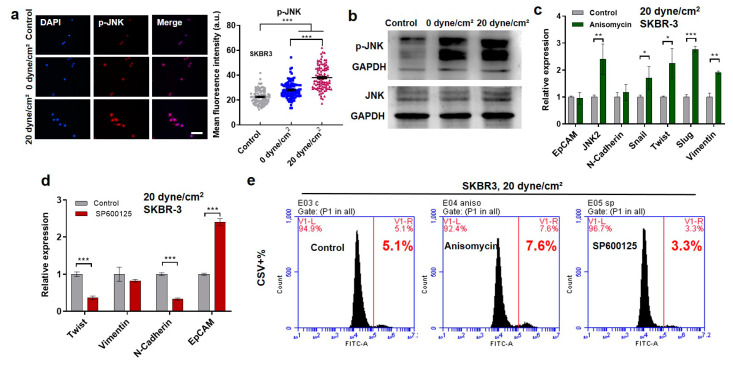
Fluid shear stress facilitates the EMT phenotype of suspended tumor cells via Jun N-terminal kinase (JNK) signaling. (**a**,**b**) Fluid shear stress activates JNK signaling. SKBR3 cells were treated under 0 and 20 dyne/cm^2^ shear stress for 12 h. The total and phosphorylated amounts of JNK were examined by immunofluorescence (**a**), scale bar in (**a**) 50 µm and western blotting (**b**). *n* > 100 cells in (**a**); (**c**) Activating JNK signaling upregulated mesenchymal genes under fluid shear stress; (**d**) Inhibiting JNK signaling downregulated mesenchymal genes and upregulated epithelial genes under fluid shear stress; (**e**) JNK signaling regulated the shear-induced EMT. SKBR3 cells were circulated under 20 dyne/cm^2^ shear stress in the presence of JNK activator Anisomycin or inhibitor SP600125 for 12 h. The gene expression was examined in (**c**) and (**d**), and the fraction of CSV+ cells was measured in (**e**). *n* = 2 in (**e**). * *p* < 0.05; ** *p* < 0.01; *** *p* < 0.001.

JNK signaling not only regulates EMT during tumor progression [37] but also responds to external mechanical stimulations [38], suggesting that fluid shear stress may induce EMT phenotype via JNK signaling. To test this idea, the activity of JNK signaling was examined after shear stress treatment. The results show that the phosphorylation of JNK signaling was higher in CTCs after the treatment by 20 dyne/cm^2^ than 0 dyne/cm^2^ shear stress in multiple breast cancer cells, including SKBR3, MDA-MB-468, and MCF-7 (Figure 3a and Figure S3a,c). The activation of JNK signaling was further confirmed by immunoblotting, while there was no obvious difference in the total level of JNK between control cells and shear-treated cells (Figure 3b). To explore the role in shear-induced EMT, JNK signaling was modulated when breast cancer cells were exposed to fluid shear stress. Anisomycin, a JNK activator, significantly upregulated the expressions of mesenchymal markers and facilitated the EMT phenotype of suspended tumor cells in the presence of 20 dyne/cm^2^ shear stress (Figure 3c and Figure S3b,d). On the other hand, SP600125, a JNK inhibitor, enhanced the expressions of epithelial markers (EpCAM), while it suppressed the expression of mesenchymal markers (Twist, N-cadherin) under fluid shear stress (Figure 3d and Figure S3b,d). Importantly, silencing JNK signaling decreased the fraction of CSV+ subpopulation in the cells treated by 20 dyne/cm^2^ shear stress (Figure 3e), while activating this pathway increased this fraction. These findings demonstrate that inhibiting/activating JNK signaling suppresses/facilitates the shear-induced EMT of breast CTCs, suggesting that fluid shear stress induces EMT in suspended CTCs via JNK signaling.

### 2.4. Fluid Shear Flow Facilitates the Transition of Epithelial Tumor Cells into Mesenchymal Phenotype and Enriches Pre-Existing Mesenchymal CTCs

**Figure 4:**
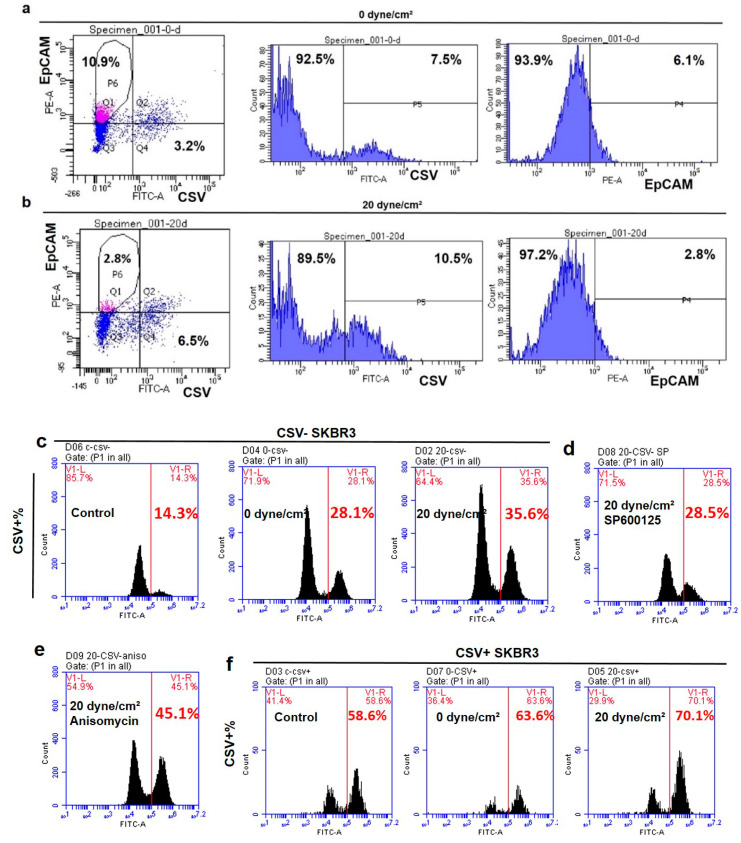
Fluid shear stress facilitates the transition of epithelial circulating tumor cells (CTCs) into mesenchymal status. (**a**–**c**) The transition of epithelial tumor cells into mesenchymal phenotype under fluid shear stress. EpCAM+/CSV− and CSV− cells were sorted out from untreated SKBR3 cells and then exposed to 0 and 20 dyne/cm^2^ shear stress for 12 h. The percentages of EpCAM+/CSV−, CSV+, and EpCAM+ subpopulations were measured by flow cytometry. (**d**,**e**) The role of JNK signaling in the transition of epithelial CTCs into mesenchymal status. The sorted CSV− cells were exposed to 20 dyne/cm^2^ shear stress in the presence of SP600125 or Anisomycin. (**f**) The enrichment of pre-existing mesenchymal tumor cells by shear stress. The sorted CSV+ cells were exposed to 0 and 20 dyne/cm^2^ shear stress as well as cultured in petri dishes for 12 h, when the fraction of CSV+ cells was analyzed. All the flow cytometry figures were representative of two independent experiments.

Our findings demonstrated that fluid shear stress promotes EMT in suspended CTCs, which may be through the transition of epithelial CTCs into mesenchymal cells and/or enrichment of pre-existing mesenchymal cells. To test this possibility, the EpCAM+/CSV− and EpCAM−/CSV+ cell subpopulation fractionated by fluorescence-activated cell sorting (FACS) were adopted to represent epithelial and mesenchymal CTCs and then treated under 0 and 20 dyne/cm^2^ shear stress, respectively. The results showed that the percentage of EpCAM+/CSV− cells was ~11% after the treatment by 0 dyne/cm^2^ shear stress, which was decreased to ~3% after the treatment by 20 dyne/cm^2^ shear stress (Figure 4a,b). Compared to 0 dyne/cm^2^ shear stress, the percentage of CSV+ cells was increased from 7.5% to 10.5% under 20 dyne/cm^2^ shear stress, while the percentage of EpCAM+ cells was reduced from ~6% to ~3%. Importantly, when the CSV− cell subpopulation was exposed to the suspension condition (0 dyne/cm^2^ shear stress), the percentage of CSV+ cells was increased to ~28% from ~14% for the cells cultured in petri dishes, and further elevated to ~36% under 20 dyne/cm^2^ shear stress (Figure 4c). Notably, inhibiting JNK signaling suppressed the transition of CSV− into CSV+ cells from ~36% to ~28%, which was similar to the CSV+ level under the treatment of 0 dyne/cm^2^ shear stress (Figure 4d). Activating this pathway facilitated the induction of EMT and increased the percentage of CSV+ cells to ~45% in suspended epithelial CTCs (Figure 4e). On the other hand, when culturing CSV+ cells in petri dishes, the percentage of CSV+ cells was decreased to ~59%. The transition of CSV+ cells was partially suppressed when exposed to 0 dyne/cm^2^ shear stress, and further inhibited under 20 dyne/cm^2^ shear stress (Figure 4f), suggesting that fluid shear stress is able to maintain the pre-existing mesenchymal cells in suspension. These results demonstrate that fluid shear stress facilitates the transition of epithelial into mesenchymal cells and sustains the phenotype of the pre-existing mesenchymal subpopulation.

### 2.5. Shear-Induced EMT Promotes the Survival of Suspended CTCs in Shear Flow

**Figure 5:**
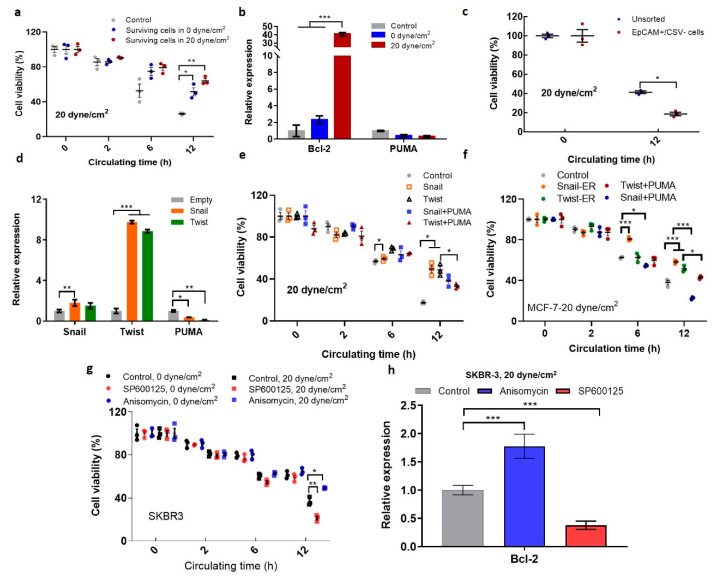
EMT phenotype promotes suspended tumor cell survival. (**a**) Tumor cells surviving fluid shear stress exhibit higher viability than untreated CTCs. SKBR3 cells were treated under 0 and 20 dyne/cm^2^ shear stress, respectively. The surviving cells were then re-circulated in 20 dyne/cm^2^ shear stress for various times. Untreated tumor cells were used as control; (**b**) Tumor cells after shear flow treatment upregulated the survival gene Bcl-2 and downregulated the pro-apoptosis gene pro-apoptosis gene p53 upregulated modulator of apoptosis (PUMA). The gene expression was tested after tumor cells were treated by 0 and 20 dyne/cm^2^ shear stress; (**c**) Epithelial CTCs exhibited low survival ability in fluid shear flow. EpCAM+/CSV− cells were sorted out via FACS, and these cells and unsorted tumor cells were treated under 20 dyne/cm^2^ shear stress for 12 h; (**d**) Overexpressing Snail or Twist downregulated PUMA. *n* = 3 independent experiments; (**e**,**f**) The induced EMT enhanced the survival of suspended tumor cells via PUMA. SKBR (**e**) and MCF-7 cells (**f**) were transfected with Snail or Twist plasmids and in the presence or absence of PUMA plasmids. These cells were then circulated under 20 dyne/cm^2^ shear stress for various times. Cell viability was measured by MTS assay; *n* = 3 independent experiments; (**g**) JNK signaling affected the survival of suspended tumor cells under shear flow; (**h**) JNK signaling regulated the expressions of Bcl-2 of suspended tumor cells under shear flow. SKBR3 cells were treated by 0 and 20 dyne/cm^2^ shear stress in the presence of Anisomycin and SP600125, respectively. Cell viability was measured in (**g**), and the gene expression was analyzed in (**h**). The statistics were conducted using ANOVA with the post hoc Tukey test in (**a**,**b**,**d**,**e,h**) and using a two-tailed Student’s *t*-test in (**c**). * *p* < 0.05; ** *p* < 0.01; *** *p* < 0.001.

It is known that EMT enhances tumor cell motility and promotes metastasis [39,40]. Our data showed that fluid shear stress facilitated the EMT phenotype of suspended tumor cells via JNK signaling. We thus further examined the influence of shear-induced EMT on the survival of suspended CTCs in fluid shear stress. The survival ability of the surviving CTCs was tested after being re-circulated under fluid shear flow. The results showed that the surviving tumor cells after the treatment by 0 and 20 dyne/cm^2^ shear stress exhibited higher viability than untreated cells (Figure 5a), suggesting that shear-induced EMT is correlated with high survival ability under shear flow. This may be explained by the upregulation of anti-apoptosis gene Bcl-2 and downregulation of pro-apoptosis gene p53 upregulated modulator of apoptosis (PUMA) (Figure 5b; Figures S4a,e,f and S5). We further explored the role of EMT in the survival of CTCs under shear stress. Importantly, the EpCAM+/CSV− CTCs exhibited significantly lower viability than unsorted tumor cells (Figure 5c), suggesting that epithelial phenotype may reduce the survival ability of CTCs in shear flow. Further, the transcription factor Snail or Twist was overexpressed to induce EMT in breast cancer cells [41,42], which significantly decreased the expressions of the pro-apoptosis gene PUMA (Figure 5d; Figure S4c,d). Notably, CTCs with the induced EMT phenotype exhibited considerably higher survival ability than control cells (Figure 5e and Figure S4b), while overexpressing PUMA partially rescued this effect (Figure 5f), suggesting that EMT enhances CTC survival in fluid shear flow possibly through the suppression of PUMA. Further, silencing JNK signaling suppressed the survival of suspended CTCs in 20 dyne/cm^2^ shear stress, while activating this pathway enhanced their survival (Figure 5g and Figure S6). This could be explained by the up-/downregulation of Bcl-2 when JNK signaling was activated/suppressed in the presence of fluid shear stress (Figure 5h). Note that JNK inactivation/activation had no obvious effect on the survival of CTCs under 0 dyne/cm^2^ shear stress (Figure 5g), suggesting that the effect of JNK signaling on tumor cell survival may be due to its influence on EMT but not the cytotoxicity of JNK modulation. These results demonstrate that shear-induced EMT phenotype promotes the survival of CTCs in blood shear flow.

### 2.6. The Expressions of EMT Markers, Bcl-2, and JUN Gene Are Correlated with Poor Survival of Breast Cancer Patients

**Figure 6:**
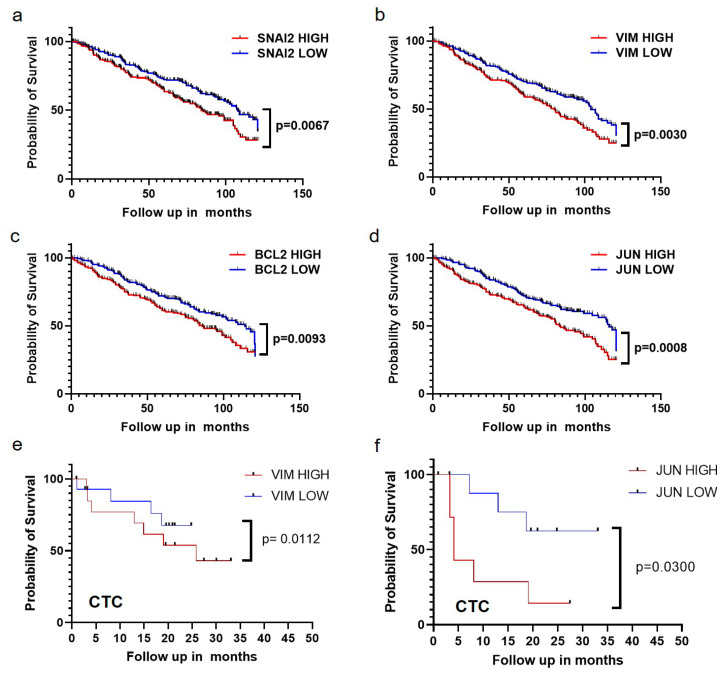
The expressions of EMT genes, Bcl-2, and JUN gene are correlated with poor diagnosis and survival of breast cancer patients. (**a**–**d**) Kaplan–Meier analysis of the relapse-free survival rate was based on the expression of Snail2 (**a**), vimentin (**b**), Bcl-2 (**c**), and JUN (**d**) in breast patients from GSE6532 (*n* = 781), GSE3494 (*n* = 315), GSE7390 (*n* = 198), and GSE1456 (*n* = 159) public databases. *n* = 1453 patients; (**e**,**f**) Kaplan–Meier analysis of the relapse-free survival rate was based on the expression of vimentin (**e**) and JUN gene (**f**) in the CTCs of breast patients from the GSE144494 database. *n* = 45 patients. The patients were split into three tertile groups in terms of gene expression. The group with high/low gene expression represented cancer patients in the highest/lowest tertile. The red and blue line represents the high and low expression of the indicated gene. The statistical analysis in the survival curves was conducted using the log–rank (Mantel–Cox) test.

Our results have demonstrated that fluid shear stress facilitates the transition of epithelial CTCs into mesenchymal status, which further promotes their survival in blood shear flow via PUMA and JNK signaling. These surviving cells may harbor the cells that can initiate the formation of metastases and eventually deteriorate patient survival. To test this possibility, we further examined the correlation between the expressions of EMT genes, Bcl-2, and JNK target gene JUN in both patient tumors and CTCs and the survival of breast cancer patients. Four databases (GSE6532, GSE3494, GSE7390, and GSE1456) from Gene Expression Omnibus (GEO) were combined to analyze the relationship between the gene expressions in patient tumors and their survival, while one database GSE144494 was utilized for the analysis of CTCs. The data showed that high expressions of EMT regulators Snail and vimentin, Bcl-2, and JUN were associated with a poor recurrence-free survival of breast cancer patients (Figure 6a–d), which may be explained by our findings that high expressions of these genes facilitated the survival of CTCs under blood shear stress that may enhance the metastatic risk and deteriorate patient survival. Further, we attempted to examine the relationship between the gene expressions in patient CTCs and the prognosis of breast cancer patients. High expressions of vimentin and JUN gene in CTCs were associated with poor patient survival (Figure 6e,f). These results suggest that the expressions of EMT genes, Bcl-2, and JNK target gene JUN are correlated with patient survival and can thus predict the prognosis of breast cancer patients.

## 3. Discussion

Hematogenous dissemination is the major route for cancer cells to metastasize to distal organs, in which they experience considerable levels of fluid shear stress. It has been demonstrated that mechanical signals, including fluid shear stress, influence the EMT phenotype of tumor cells, the majority of which have focused on tumor cells that are attached to solid substrates [26,43]. Recent findings show that fluid shear stress significantly enhances cancer stem cell-like properties but not the EMT signature of breast cancer cells in suspension [30]. Another study reports that hemodynamic shear stress facilitates the EMT-like transition and stemness acquisition of suspended cancer cells [29]. Nevertheless, the influence of fluid shear stress on the EMT phenotype of suspended CTCs and the underlying mechanisms are still not fully understood. Our study showed that blood shear stress significantly up-/downregulates mesenchymal/epithelial markers and thus enhances the mesenchymal fraction within heterogeneous CTCs. Specifically, fluid shear stress promotes the transition of epithelial CTCs into a more mesenchymal status dependent on JNK signaling and suppresses the conversion of mesenchymal cells into epithelial status. Further, the surviving cells exhibit EMT-like phenotype, and mesenchymal CTCs survive much better than epithelial cells in fluid shear flow, suggesting that fluid shear stress has the potential to enrich the pre-existing mesenchymal CTCs. These findings demonstrate that fluid shear stress promotes EMT through inducing the transition of epithelial CTCs into mesenchymal cells and selecting pre-existing mesenchymal cells. Mechanistically, fluid shear stress activates JNK signaling, which further regulates the EMT phenotype of suspended CTCs. Inhibiting/activating JNK signaling in CTCs suppresses/promotes the transition of epithelial cells into mesenchymal cells or the induction of EMT under shear flow. Although our findings demonstrated the involvement of JNK signaling in shear-induced EMT, other mechanisms cannot be excluded in this mechanoresponsive process. Note that the suspension condition (0 dyne/cm^2^) had a moderate effect on the EMT phenotype of CTCs, while high shear stress (20 dyne/cm^2^) further promoted the transition of epithelial cells into mesenchymal phenotype.

Less than 0.01% of CTCs eventually generated metastatic colonization due to various rate-limiting factors, including the survival in the vasculature. Consistently, our study, together with many others [10,29,30,33], have illustrated that fluid shear stress is one major factor that affects CTC survival in blood circulation. Nevertheless, there is still a small subpopulation of CTCs surviving shear flow that may harbor the cells with the ability to generate metastases in distal organs. Several reports have suggested that suspended CTCs may survive fluid shear stress through Lamin A/C [12], actomyosin [13,14], and ATOH8 [15]. EMT facilitates the resistance of tumor cells to anoikis or suspension conditions [21,22]. However, whether and how the shear-induced EMT phenotypic change affects the survival of suspended CTC in blood shear flow remains unclear. Our results showed that the surviving tumor cells exhibited mesenchymal phenotype via activated JNK signaling, upregulated Bcl-2, and downregulated PUMA, and survived much better than control cells in shear flow, suggesting JNK-mediated EMT is correlated with enhanced survival of suspended CTCs. The induction of EMT significantly downregulated PUMA and increased tumor cell viability, which could be blocked by activating PUMA. Activating/silencing JNK signaling promoted/suppressed EMT occurrence and Bcl-2 expression and enhanced/reduced the survival of CTCs under fluid shear stress. These findings suggest that fluid shear stress induces JNK-mediated EMT, which facilitates the survival of suspended CTCs in shear flow through PUMA and Bcl-2. Further, the high expression of EMT genes and JNK target genes is correlated with poor patient survival. These findings are consistent with the fact that tumor cells with high metastatic potential exhibit the EMT phenotype [44], possess survival advantage during the entire metastasis process [39], and mediate poor patient survival. Nevertheless, whether the surviving CTCs with shear-induced EMT phenotype can effectively re-attach to the endothelium, intravasate into the secondary tissue, and generate metastatic tumors remains unclear. Several other studies reported that the number of EpCAM^high^ but not EpCAM^low^ CTCs in periphery blood is correlated with poor overall survival in castration-resistant prostate cancer patients and metastatic lung cancer patients [45,46]. These seemingly inconsistent findings may be partially due to the multiplex roles of EpCAM or other EMT regulators since it not only serves as an epithelial marker but also has been implicated in the stemness and tumorigenicity of cancer cells [47].

Therefore, our findings have demonstrated the dual effects of fluid shear stress on CTCs. One is that fluid shear flow eliminates the majority of CTCs during hematogenous dissemination, which serves as an important rate-limiting factor and contributes to metastasis inefficiency. The other one is to induce EMT in suspended CTCs via JNK signaling, which enhances their survival in the vasculature and may possibly favor the subsequent generation of metastases and thus deteriorate patient survival. Since blood shear stress is able to influence the EMT phenotype of CTCs and transit EpCAM+ cells into EpCAM− cells with an elevated ability to survive under shear flow, it is thus likely to observe both epithelial and mesenchymal phenotypes within CTCs, which is consistent with the previous findings [48,49] and adds another layer of heterogeneity to the CTC population. However, current techniques for CTC harvest mainly rely on cell surface markers, especially EpCAM [48]. Therefore, the EpCAM-based strategies cannot enrich those EpCAM− tumor cells from the peripheral blood, which may not recapitulate the complexity of CTCs and need to be revisited in the future. In addition, CTCs in the peripheral blood of cancer patients are very rare (1–10 CTCs per ml blood) [2]. Single-cell suspensions of cancer cell lines cannot fully represent but recapitulate certain aspects of bona fide CTCs, and thus serve as an alternative model of CTCs. Nevertheless, more efforts are needed to rigorously test our current findings using patient-derived CTCs in the future.

## 4. Materials and Methods

### 4.1. Shear Stress Treatment 

The in vitro circulation system mainly included a peristaltic pump (P-230, Harvard Instruments, Holliston, MA, USA), a silicone microtube (diameter 0.51 mm, length 1.5 m), and a syringe used as a reservoir for the cell solution, which could simulate fluid shear stress in the blood circulation by producing pulsating flow. In accordance with Poiseuille’s law, the wall shear stress τ (dyne/cm^2^) in the tubing was calculated by τ = 4µQ/(πR^3^), where Q is the flow rate (from 0.001 to 230 mL/min) and µ is the liquid dynamic viscosity (0.01 dyne/cm^2^ for cell culture media), R is the radius of the tube (0.255 mm). The entire system was sterilized with 75% ethanol and then rinsed with 4 mL of phosphate-buffered saline (HyClone Laboratories, South Logan, UT, USA) before the experiment. To avoid the attachment of suspended tumor cells to the tubes and syringes, the system was treated with 4 mL of 1% bovine serum albumin (VWR Life Science, Radnor, PA, USA). In the process of the experiment, 2 mL of cell suspension (2 × 10^5^ cells/mL) was added into the circulation system and subjected to different magnitudes of shear stress for different durations in the cell culture incubator at 37 °C and 5% CO_2_.

### 4.2. Cell Culture and Plasmid Transfection

Human breast cancer cell lines SKBR3, MDA-MB-468, MCF-7, and MDA-MB were purchased from ATCC. Cells were cultured in petri dishes with Dulbecco’s Modified Eagle Medium (DMEM; HyClone, Logan, UT, USA) supplemented with 10% fetal bovine serum (HyClone, South Logan, UT, USA), and 1% penicillin/streptomycin (Gibco, Co Dublin, Ireland) in an atmosphere of 5% CO_2_ at 37 °C. Cells were passaged every 2–3 days using 0.25% Trypsin (Gibco, Co Dublin, Ireland). For plasmid transfection, pWZL Blast Twist ER, pWZL Blast Snail ER, and empty vector pWZL Blast GFP were obtained from Addgene (Cambridge, MA, USA). These plasmids were transfected into cells using Lipofectamine 3000 Reagent (Thermo Fisher, Waltham, MA, USA). The validation of transfection was conducted using quantitative RT-PCR analysis.

### 4.3. MTS

Cell viability was measured by the MTS method following the manufacturer’s instructions (Promega, Madison, WI, USA). Briefly, 100 µL of cell suspension was collected from the circulation system and then added to 1 well of a 96-well plate. After incubation for 12 h, 20 µL of sterile CellTiter 96 aqueous solution (5 mg/mL; Promega, Madison, WI, USA) was added to each well, and the plate was incubated for 4 h at 37 °C. The absorbance of the cell solution was measured at 490 nm using a Benchmark Plus microplate reader (Bio-Rad, Hercules, CA, USA).

### 4.4. Annexin V Assay

Cell apoptosis was measured through the Annexin V-fluorescein isothiocyanate (FITC) Apoptosis Staining/Detection kit (Abcam, Cambridge, UK) and BD Accuri C6 Flow Cytometer (BD Biosciences, San Jose, CA, USA). One hundred thousand cells were collected and resuspended in 500 µL of binding buffer (ABCAM, Cambridge, UK). Five microliters of Annexin V-FITC and 5 µL of propidium iodide (PI) were added and incubated with the cells for 5 min at 4 °C without light. The flow cytometer was used to fractionate at least 10,000 cells based on the fluorescence of FITC signals and the PI phycoerythrin signals. The results were analyzed with BD Accuri C6 software.

### 4.5. Quantification of Cell Spreading and Aspect Ratio

After shear stress treatment, tumor cells were seeded on polyacrylamide gels with different stiffness (0.6, 1.5, and 5 kPa) for 2, 4, 8, and 12 h. Before capturing images by the inverted microscope (Nikon, Tokyo, Japan), unattached cells were removed by washing with PBS gently. At least 60 cells/condition were imaged, from which cell spreading and aspect ratios were quantified by the ImageJ software (NIH, Bethesda, MD, USA).

### 4.6. Flow Cytometry and Fluorescence-Activated Cell Sorting (FACS)

The antibodies EpCAM-Phycoerythrin (PE) (Abcam, Cambridge, UK), EpCAM-FITC (Abcam, Cambridge, UK), and cell-surface vimentin (CSV) (Abnova, Taipei, Taiwan) were diluted in phosphate-buffered saline (PBS) containing 2% fetal bovine serum (FBS). The cells were stained with the corresponding antibody solution in the dark for at least 30 min and washed with PBS 3 times before being resuspended in PBS containing 2% FBS. These cells were then analyzed by a BD FACSVia™ flow cytometer (BD Biosciences, San Jose, CA, USA) and sorted using a BD FACSAria III cell sorter (BD Biosciences, San Jose, CA, USA).

### 4.7. RT-PCR Analysis 

Total mRNAs were extracted by an Aurum Total RNA Mini Kit (Bio-Rad, Hercules, CA, USA), and complementary DNA was synthesized with a RevertAid First Strand cDNA Synthesis Kit (Thermo Fisher Science, Waltham, MA, USA). Quantitative RT-PCR was performed using the Forget-Me-Not EvaGreen qPCR Master Mix with Rox (Biotium, Fremont, CA, USA) and a CFX96 Real-Time PCR detection system (Bio-Rad, Hercules, CA, USA). The sequences of all primers were obtained from the National Centre for Biotechnology Information database and are listed in Table S1. For data analysis, the expression of all genes was normalized using the ∆∆cycle threshold method against human glyceraldehyde 3-phosphate dehydrogenase.

### 4.8. Immunofluorescence

After shear stress or suspension treatment, cancer cells were seeded on coverslips and fixed with 4% Paraformaldehyde Solution (PFA) (Thermo Scientific™, Waltham, MA, USA) for 20 min. The cells were then blocked and permeabilized with 0.1% Triton X-100 (SAFC) in 1% BSA for 1 h at room temperature. These treated cells were incubated with the primary antibody: EpCAM-Phycoerythrin (PE) (Abcam, Cambridge, UK); Twist (Abcam, Cambridge, UK); E-cadherin (Abcam, Cambridge, UK); N-cadherin (Abcam, Cambridge, UK); Bcl-2 (Abcam, Cambridge, UK); PUMA (Abcam, Cambridge, UK) at 4 °C overnight and then stained with the secondary antibody: Goat Anti-Mouse IgG H&L (Alexa Fluor^®^ 488) (Abcam, Cambridge, UK); Goat Anti-Rabbit IgG H&L (Alexa Fluor^®^ 594) (Abcam, Cambridge, UK) at room temperature for 1 h. Cells were rinsed with PBS 3 times for each step, and the nucleus was counterstained by ProLong Gold Antifade Mountant with 4′,6-diamidino-2-phenylindole (DAPI) (Thermo Fisher Scientific, Waltham, MA, USA). At least 100 cells/condition were imaged by the fluorescent microscope (Nikon, Tokyo, Japan) using PE, FITC, and DAPI channels, respectively. Fluorescence intensity was analyzed using ImageJ (NIH, Bethesda, MD, USA).

### 4.9. Kaplan–Meier Survival Analysis

The GSE6532, GSE3494, GSE1456, and GSE7390 microarray expression profile and raw data and gene probe platforms were collected from the Gene Expression Omnibus database (GEO, http://www.ncbi.nlm.nih.gov/geo/). These included information from 781, 315, 159, and 198 patients, respectively. All these databases used relapse-free survival (RFS) to record patient survival time, adopted the same probe platform HG-U133A, and defined patient death as one event. Therefore, they could be merged for the following analysis. The gene expression was analyzed using an R/Bioconductor. Briefly, all databases were combined by the R package scMerge. Then, the probe intensities were extracted from Affymetrix CEL files with the background corrected, and all data were log2-transformed, normalized, and summarized to the probe-set expression using the sva and limma software package and affy package with the default settings. The HG-U133A annotation package was used to map the probe-set to the Entrez gene ID. The average gene expression was used when multiple probe sets were mapped to the same Entrez gene ID. Finally, the gene expression matrix was obtained from the four databases. All R language packages were downloaded from https://bioconductor.org/. The clinical gene expression data and patient RFS were combined and analyzed using GraphPad Prism 8.0. In addition, the database GSE144494 included RNA-seq profiles of 135 freshly isolated single CTCs or CTC-clusters from 45 women with hormone receptor-positive metastatic breast cancer. The relationship between the prognosis of breast cancer patients and the expressions of EMT- and JNK-related genes was analyzed by GraphPad Prism 8.0. The statistical analysis was conducted with the log–rank (Mantel–Cox) test.

### 4.10. Statistical Analysis

All the data were shown as mean ± SEM (standard error of the mean). For the comparisons between two conditions and among three or more conditions, a two-tailed Student’s *t*-test or ANOVA analysis was conducted, respectively. The post hoc Tukey or Bonferroni test was adopted for the comparisons with equal or unequal sample sizes, respectively. The statistical analysis in the survival curves was conducted using the log–rank (Mantel–Cox) test. *, *p* < 0.05, **, *p* < 0.01 ***, *p* < 0.001.

## 5. Conclusions

Our study has demonstrated that fluid shear stress in blood circulation not only eliminates the majority of suspended CTCs but also facilitates the EMT phenotype in the surviving tumor cells through transiting epithelial tumor cells into mesenchymal status via JNK signaling and maintaining the phenotype of pre-existing mesenchymal cells. Importantly, the shear-induced EMT promotes the survival of suspended CTCs in blood shear stress by suppressing the expression of PUMA and activating JNK signaling. Further, the expressions of EMT genes and JNK gene JUN were correlated with poor patient survival. Our findings unveiled the dual effects of fluid shear stress in blood circulation on CTCs and shed new insight into the roles of mechanics in tumor metastasis.

## Supplementary Materials

Supplementary materials can be found at https://www.mdpi.com/1422-0067/21/21/8115/s1, There are six additional figures and one additional table in the supplementary information.

## Abbreviations

CTCs	Circulating tumor cells
EMT	Epithelial–mesenchymal transition
CSV	Cell surface vimentin
EpCAM	Epithelial cell adhesion molecule
