# Supplementary material for: Fluid Shear Stress Induces EMT of Circulating Tumor Cells via JNK Signaling in Favor of Their Survival during Hematogenous Dissemination
Source: Int J Mol Sci. 2020 Oct 30;21(21):8115. doi: 10.3390/ijms21218115 (PMC7663710; doi:10.3390/ijms21218115)
Supplement: Supplementary file 1 [file ijms-21-08115-s001.pdf]

Fluid shear stress induces EMT of circulating tumor cells via JNK signaling in favour of their survival during hematogenous dissemination

Ying Xin[1,2#], Keming Li[1,2#], Mo Yang[2], Youhua Tan[1,2*]

[1] The Hong Kong Polytechnic University Shenzhen Research Institute, Shenzhen, China
[2] Department of Biomedical Engineering, The Hong Kong Polytechnic University, Hong Kong, China
#: Contributed equally
* Corresponding author: Youhua TAN (youhua.tan@polyu.edu.hk)

Supplementary Materials

Table S1

Genes		Quantitative RT-PCR
E-cadherin	5′ primer	TGCCCAGAAAATGAAAAAGG
	3′ primer	GTGTATGTGGCAATGCGTTC
Snail	5′ primer	CCTCCCTGTCAGATGAGGAC
	3′ primer	CCAGGCTGAGGTATTCCTTG
Twist	5′ primer	GGAGTCCGCAGTCTTACGAG
	3′ primer	TCTGGAGGACCTGGTAGAGG
Vimentin	5′ primer	ACTCCCTCTGGTTGATAC
	3′ primer	ATCGTGATGCTGAGAAGT
Bcl-2	5′ primer	GTCATGTGTGTGGAGAGCGTCAACC
	3′ primer	CCAGGGCCAAACTGAGCAGAGTC
Slug	5′ primer	GGGGAGAAGCCTTTTTCTTG
	3′ primer	TCCTCATGTTTGTGCAGGAG
PUMA	5′ primer	GACGACCTCAACGCACAGTA
	3′ primer	CTAATTGGGCTCCATCTCG
GAPDH	5′ primer	GCGACACCCACTCCTCCACCTTT
	3′ primer	TGCTGTAGCCAAATTCGTTGTCATA

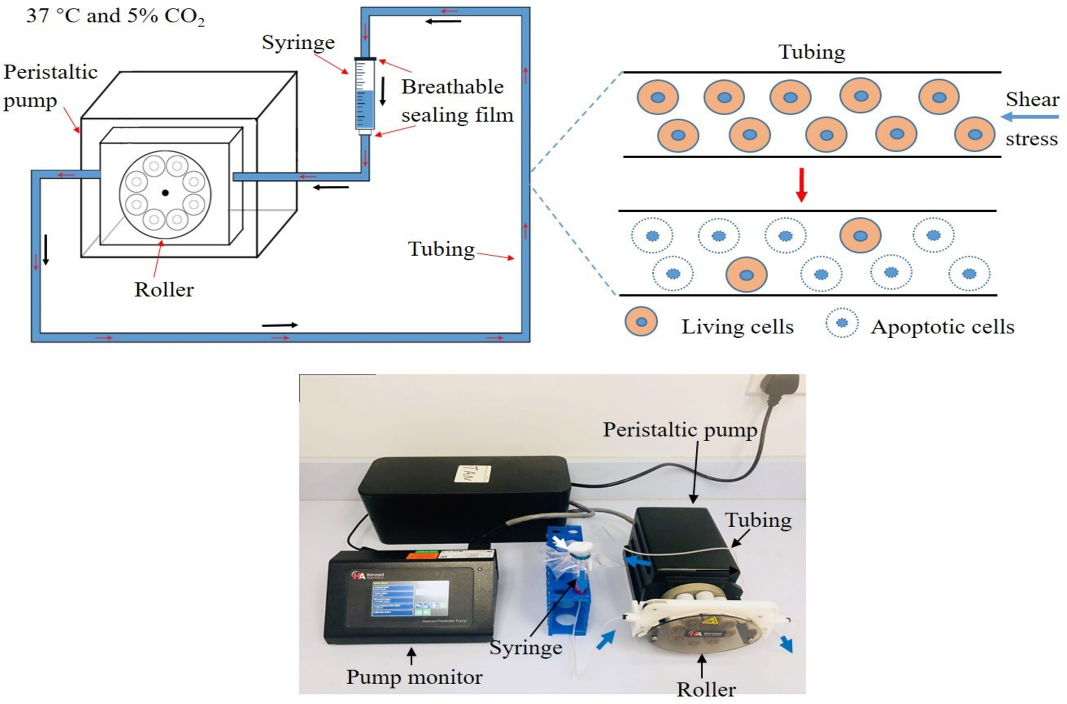

Figure S1. The schematic of the circulatory microfluidic system. The system was composed of a peristaltic pump (P-230, Harvard Apparatus), a silicone micro-tubing (0.51 mm in diameter and 1.5 m in length), and a syringe as cell solution reservoir. This system could generate pulsatile flow, which mimicked the hemodynamic shear stress in blood circulation. Tumor cells in suspension were treated by various magnitudes of shear stress and circulation duration.

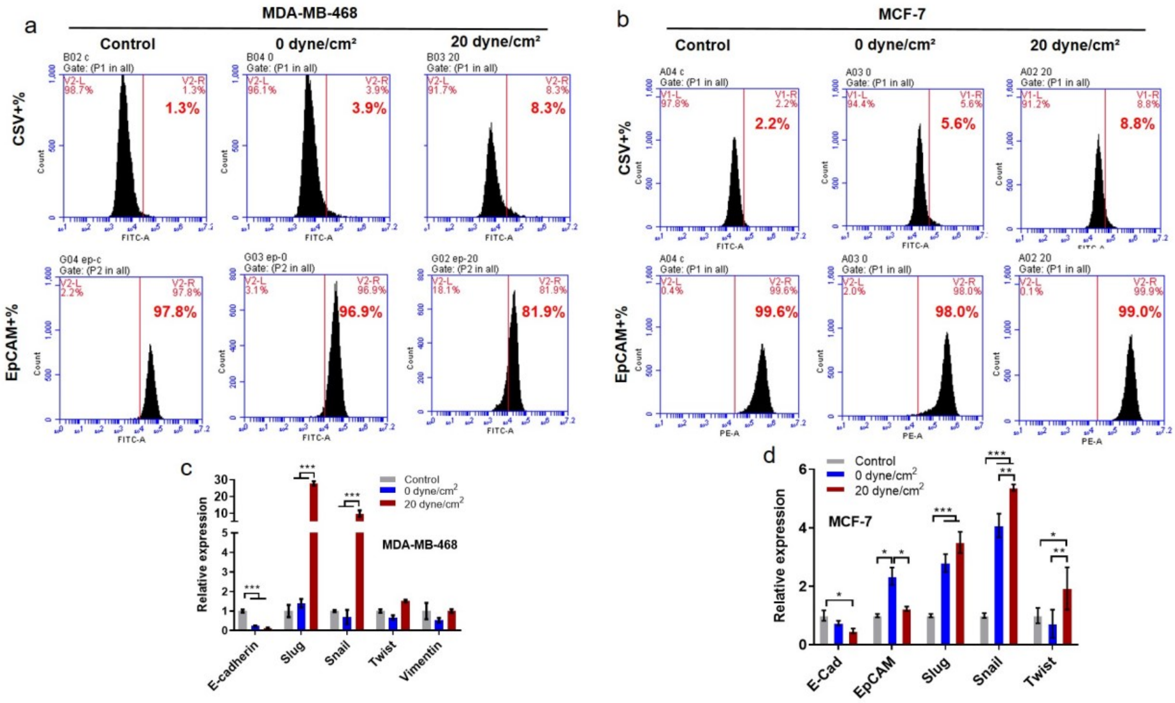

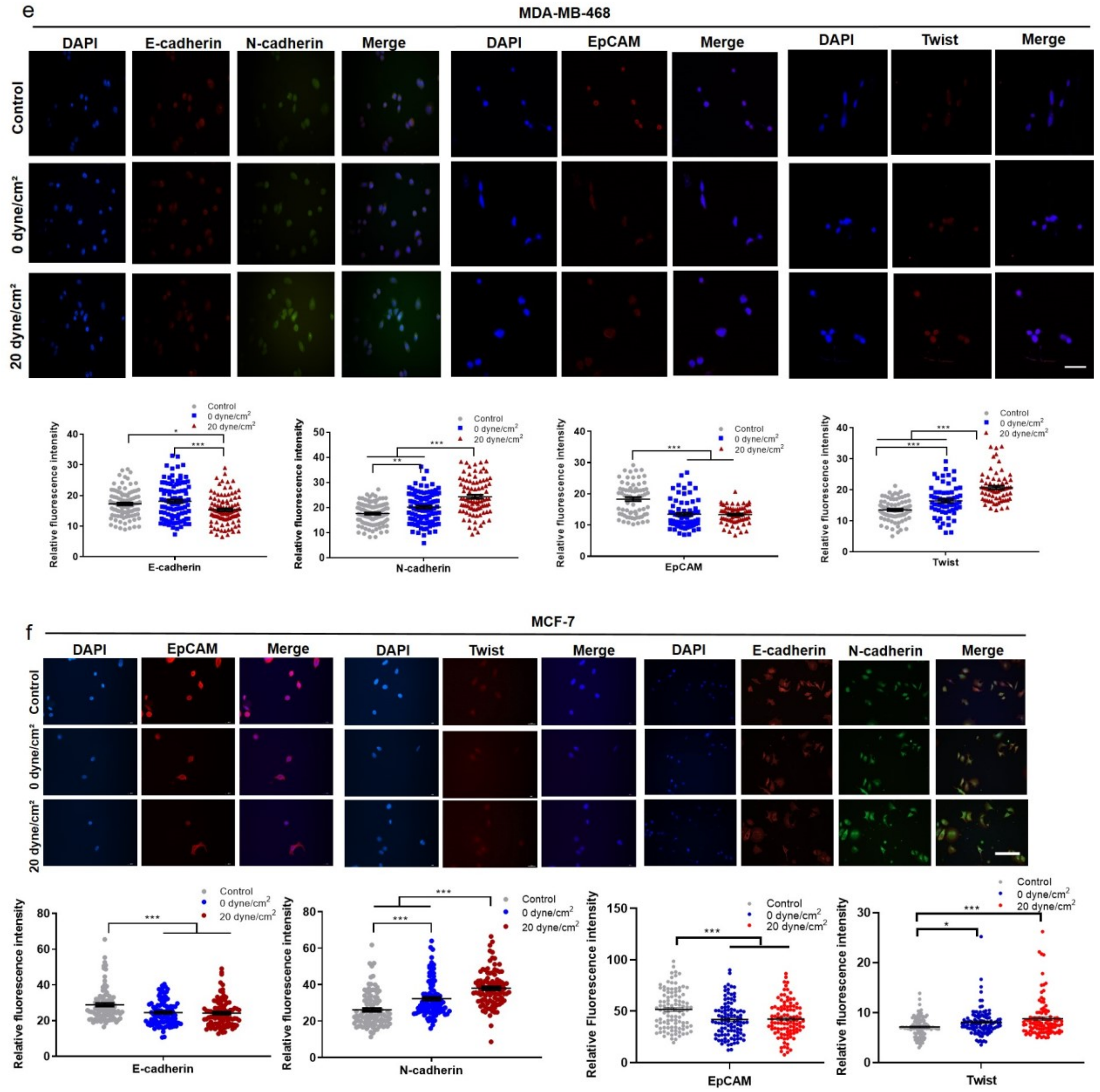

Figure S2. Fluid shear stress facilitates EMT in suspended CTCs. (a, b) Fluid shear stress increases the CSV+ fraction while decreases EpCAM+ fraction. Suspended tumor cells MDA-MB-468 (a) and MCF-7 (b) were treated with 20 dyne/cm² shear stress for 12 h. The fractions of CSV+ and EpCAM+ cells were determined by flow cytometry. (c, d) Tumor cells upregulate the EMT-related genes and downregulate epithelial marker E-Cadherin after shear flow treatment. Tumor cells were treated similarly as in (a, b) and the gene expressions were measured by RT-PCR. Cells cultured in Petri dishes were used as control. (e, f) Immunofluorescence imaging of epithelial markers E-Cadherin/Ep-CAM and mesenchymal maker Twist and N-Cadherin in breast cancer cells after shear stress treatment. MDA-MB-468 (e) and MCF-7 (f) cells were treated under 0 and 20 dyne/cm² shear stress for 12 h and then plated on glass for 12 h, when EMT-related markers were measured. The nucleus was counterstained with 4′6- diamidino-2-phenylindole (DAPI). Scale bars, 100μm. At least 100 cells were measured for each condition: n=3 independent experiments. The statistics were conducted using ANOVA with the post hoc Bonferroni test in (c-f). *p < 0.05; **p<0.01; ***p < 0.001.

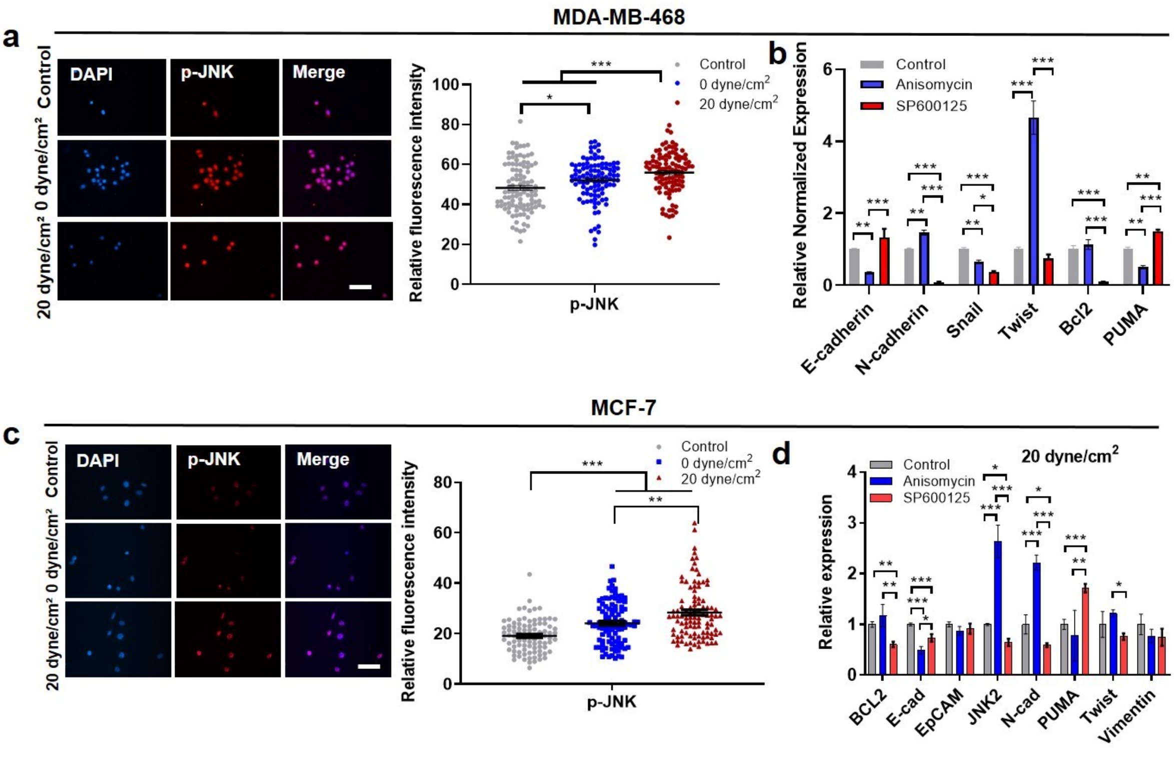

Figure S3. Fluid shear stress facilitates JNK-mediated EMT. (a, c) Fluid shear stress activates JNK signaling in suspended tumor cells. MDA-MB-468 (a) and MCF-7 (c) cells were treated under 0 and 20 dyne/cm² shear stress for 12 h. The JNK phosphorylation of these cells was measured by immunofluorescence staining. Scale bars, 100 µm. (b, d) JNK signaling regulates shear-induced EMT. MDA-MB-468 (b) and MCF-7 (d) cells were treated under 0 and 20 dyne/cm² shear stress in the presence of Anisomycin and SP600125 for 12 h, respectively. The EMT genes were then measured.

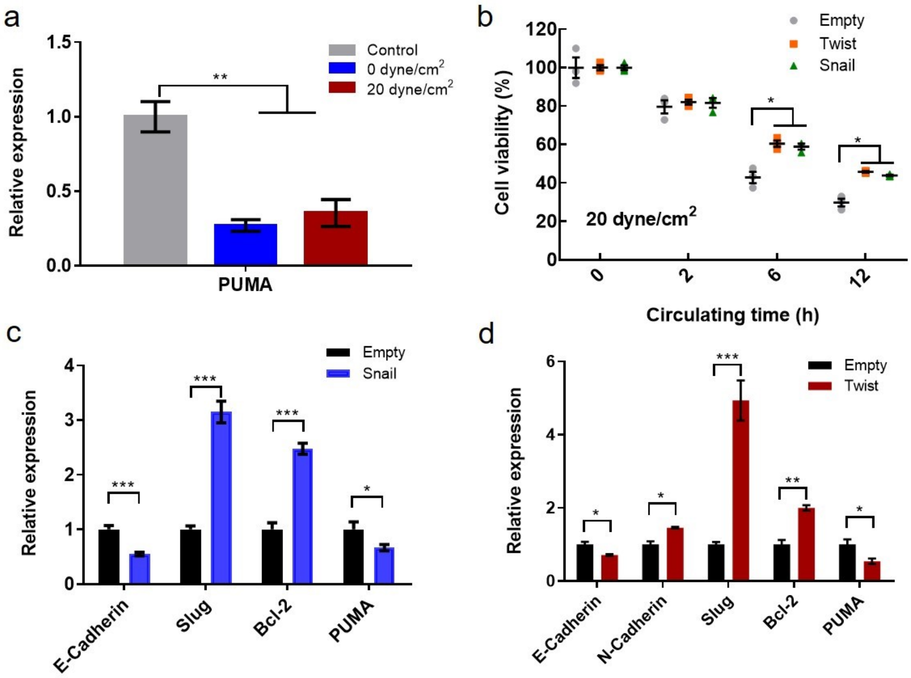

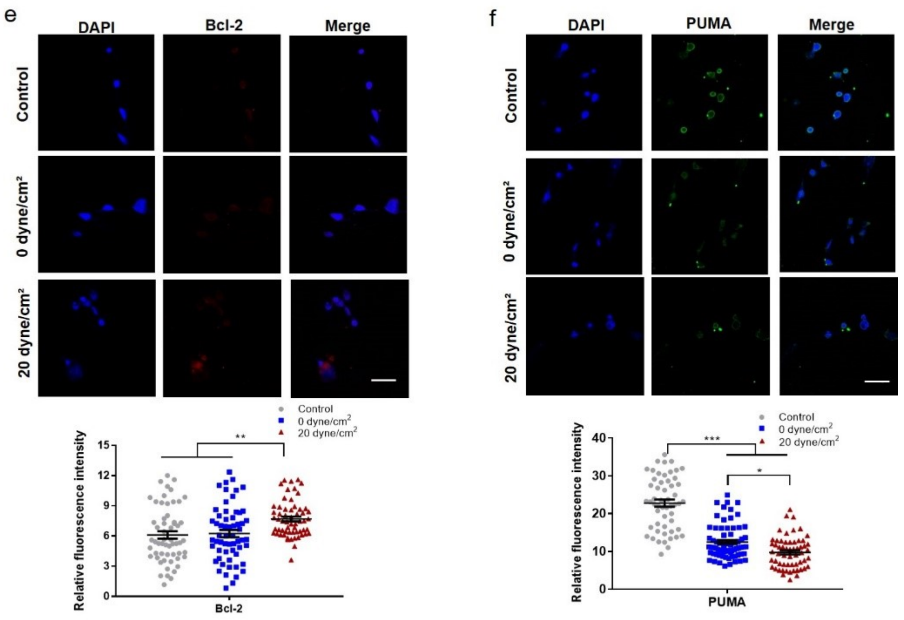

Figure S4. (a) Tumor cells MDA-MB-468 downregulate the pro-apoptosis gene PUMA after shear flow treatment. Cells cultured in Petri dishes were used as control. $^{**}p < 0.01$. (b) Overexpressing Snail or Twist enhances the viability of suspended breast tumor cells in fluid shear flow. MDA-MB-468 cells were transfected with Snail and Twist plasmids and then circulated under 20 dyne/cm^2 shear stress. Cell viability was measured by MTS assay; n=3 independent experiments. (c, d) Overexpressing Snail or Twist upregulates Bcl-2 and downregulates PUMA. n=3 independent experiments. (e, f) Fluid shear stress up-regulates Bcl-2 while down-regulates PUMA. Immunofluorescence imaging of survival gene Bcl-2 (e) and pro-apoptosis gene PUMA (f) in breast cancer cells SBKR3 after shear stress treatment. SKBR3 cells were treated under 0 and 20 dyne/cm^2 shear stress for 12 h and then plated on glass for 12 h, when the survival-related markers were stained. The nucleus was counterstained with 4’6-diamidino-2-phenylindole (DAPI). Scale bars, 100μm. At least 100 cells were measured for each condition: n=3 independent experiments. The statistics were conducted using ANOVA with the post hoc Tukey test in (a), (b), (e), (f) and two-tailed Student t-test in (c) and (d). $^{*}p < 0.05$; $^{**}p < 0.01$; $^{***}p < 0.001$.

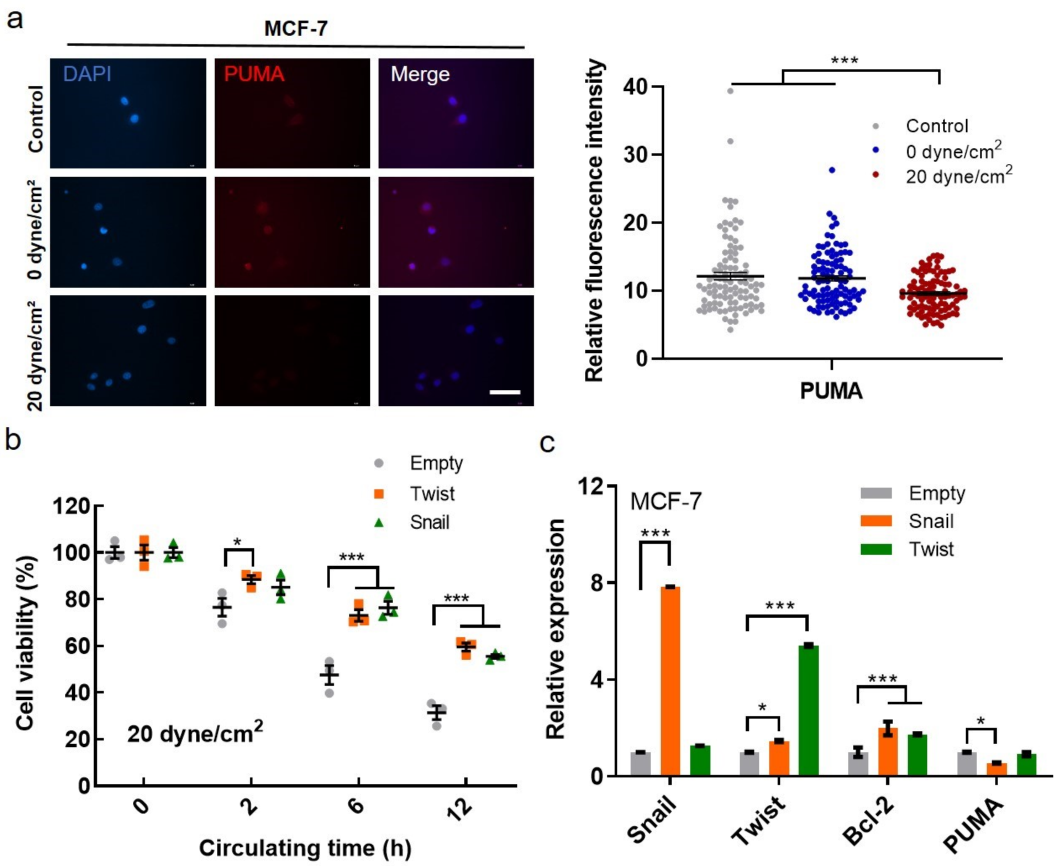

Figure S5. EMT facilitates tumor cell survival under shear stress by up-regulating Bcl-2 and down-regulating PUMA. (a) Fluid shear stress suppresses PUMA expression in suspended tumor cells. MCF-7 cells were treated under 0 and 20 dyne/cm² shear stress for 12 h. PUMA was stained in the treated cells. Scale bars, 100μm. At least 100 cells were measured for each condition. (b) Overexpressing Twist or Snail enhances tumor cell survival in shear flow. (c) Overexpressing Twist or Snail up-regulates Blc-2 and down-regulates PUMA.

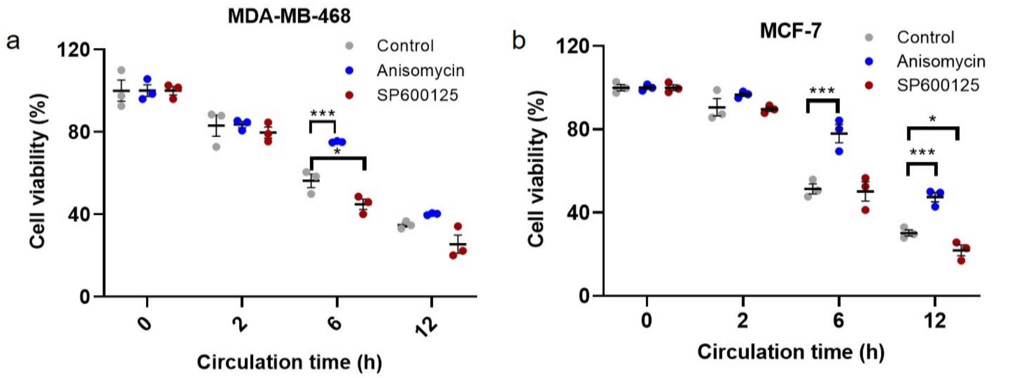

Figure S6. JNK signaling influences the survival of suspended CTCs under fluid shear stress. MDA-MB-468 (a) and MCF-7 (b) cells were circulated under 20 dyne/cm² shear stress in the presence of Anisomycin and SP600125 for various durations, respectively, when cell viability was measured by MTS assay.
